# Verification of the ProPneumo-1 assay for the simultaneous detection of *Mycoplasma pneumoniae *and *Chlamydophila pneumoniae *in clinical respiratory specimens

**DOI:** 10.1186/1476-0711-8-10

**Published:** 2009-03-10

**Authors:** Rachel R Higgins, Ernesto Lombos, Patrick Tang, Karl Rohoman, Anne Maki, Shirley Brown, Frances Jamieson, Steven J Drews

**Affiliations:** 1 Ontario Public Health Laboratories, Ministry of Health and Long-Term Care, Toronto, Ontario, Canada; 2 Department of Pathobiology and Laboratory Medicine, University of Toronto, Toronto, Ontario, Canada; 3 Mount Sinai Hospital, Toronto, Ontario, Canada

## Abstract

**Background:**

*Mycoplasma pneumoniae *and *Chlamydophila pneumoniae *are major causes of lower and upper respiratory infections that are difficult to diagnose using conventional methods such as culture. The ProPneumo-1 (Prodesse, Waukesha, WI) assay is a commercial multiplex real-time PCR assay for the simultaneous detection of *M. pneumoniae *and/or C. *pneumoniae *DNA in clinical respiratory samples.

**Objective:**

The aim of this study was to evaluate the sensitivity and specificity of the ProPneumo-1, a newly developed commercial multiplex real-time PCR assay.

**Methods:**

A total of 146 clinical respiratory specimens, collected from 1997 to 2007, suspected of *C. pneumoniae *or *M. pneumoniae *infections were tested retrospectively. Nucleic acid was extracted using an automated NucliSense easyMag (bioMerieux, Netherlands). We used a "Home-brew" monoplex real-time assay as the reference method for the analysis of *C. pneumoniae *and culture as the reference method for the analysis of *M. pneumoniae*. For discordant analysis specimens were re-tested using another commercial multiplex PCR, the PneumoBacter-1 assay (Seegene, Korea).

**Results:**

Following discordant analysis, the sensitivity of the ProPneumo-1 assay for pathogens, *C. pneumoniae *or *M. pneumoniae*, was 100%. The specificity of the ProPneumo-1 assay, however, was 100% for *C. pneumoniae *and 98% for *M. pneumoniae*. The limits of detection were 1 genome equivalent (Geq) per reaction for pathogens, *M. pneumoniae *and *C. pneumoniae*. Due to the multipex format of the ProPneumo-1 assay, we identified 5 additional positive specimens, 2 *C. pneumoniae *in the *M. pneumoniae*-negative pool and 3 *M. pneumoniae *in the *C. pneumoniae*-negative pool.

**Conclusion:**

The ProPneumo-1 assay is a rapid, sensitive and effective method for the simultaneous detection of *M. pneumoniae *and *C. pneumoniae *directly in respiratory specimens.

## Background

*Mycoplasma pneumoniae *and *Chlamydophila pneumoniae *are major causes of lower and upper respiratory tract infection in adults and children [1-3] with high incidence occurring among schoolchildren [4,5]. Both pathogens are difficult to diagnose using conventional methods such as culture and serology. Furthermore, respiratory infection may be caused by one of several bacterial or viral pathogens that share similar symptoms and clinical features. To overcome difficulties in the detection of these pathogens, PCR assays have been developed for the rapid detection and identification of pathogens directly in respiratory samples [6]. PCR assays are widely used in the clinical arena with real-time PCR slowly replacing conventional PCR assays. Several "home-brew" single target (monoplex) assays have been utilized for the detection of numerous pathogens, including *M. pneumoniae *[7-9]. However, there are multiple regulatory issues with the use of "home-brew" assays and controversies exist as to their use in a diagnostic laboratory setting. As commercial assays become more available, there is a growing movement to replace "home-brew" assays with commercially available products [10,11]. As well, the monoplex PCR format is being replaced by a multiplex one. The aim of this study was to evaluate the analytical sensitivity and the diagnostic specificity and sensitivity of the ProPneumo-1 assay, a newly developed commercial multiplex real-time PCR assay for the simultaneous detection of *M. pneumoniae *and/or *C. pneumoniae *DNA in clinical respiratory specimens.

## Methods

### Specimen collection and analysis by the reference method

Historically, suspected *C. pneumoniae *infections were tested using a "home-brew" single target monoplex real-time PCR assay [12] and suspected *M. pneumoniae *infections was tested using culture. As shown in Table 1, we examined a total of 146 clinical respiratory specimens; of which 58 were previously tested for *C. pneumoniae *using a "home-brew" real-time PCR and 88 were previously tested for *M. pneumoniae *using culture. Specimens were collected in Ontario, between 1997 and 2007, from patients with suspected *C. pneumoniae *or *M. pneumoniae *infections. The types of specimen submitted for testing at the Central Public Health Laboratory in Toronto, Ontario, including patient's sex and age are described in Table 2 for *M. pneumoniae*, and 2B for *C. pneumoniae*. Specimens and extracted nucleic acid were stored at -80°C until use.

**Table 1:** Summary of results for the evaluation of the ProPneumo-1 multiplex real-time PCR assay

	***C. pneumoniae***		***M. pneumoniae***	
	Monoplex	ProPneumo-1	Culture	ProPneumo-1

Positive specimens	11	14	42	41

Negative specimens	47	43	46	48

Total	58	57	88	89

Note: The ProPneumo-1 assay detected 3 positive *M. pneumoniae *specimens in the *C. pneumoniae*-negative pool and 2 positive C. *pneumoniae *specimens in the *M. pneumoniae*-negative pool. Accordingly, 57 and 89 specimens were resolved for C. *pneumoniae *and *M. pneumoniae*, respectively by the ProPneumo-1 assay compared to 58 and 88 by the Monoplex assay and culture, respectively. The total number of specimens tested remains 146.

**Table 2:** Overview of test results for a subset of *M. pneumoniae *patients

**Patient No**	**Sex**	**Age**	**Specimen**	**Pathogen**	**Culture**	**Prodesse** **PP-1**	**Seegene** **PB-1**
01	M	0.75	ETA	Mpn	+	+	+

02	M	32	NPS	Mpn	+	+	+

03	F	36	Sputum	Mpn	+	+	+

04	F	40	ETA	Mpn	+	+	+

05	M	26	Sputum	Mpn	+	+	+

06	F	56	Sputum	Mpn	+	+	+

07	F	72	Sputum	Mpn	+	+	+

08	F	26	BR wash	Mpn	+	+	+

09	F	7	TS	Mpn	+	+	+

10	M	NA	BAL	Mpn	+	+	+

11	F	11	NPS	Mpn	+	+	+

12	M	9	NPS	Mpn	+	+	+

13	NA	NA	NA	Mpn	+	+	+

14	M	3	NPS	Mpn	+	+	+

15	M	3	NPS	Mpn	+	+	+

16	M	6	NPS	Mpn	+	+	+

17	M	2	NPA	Mpn	+	+	+

18	M	13	NPS	Mpn	+	+	+

19	M	13	Sputum	Mpn	+	+	+

20	F	17	NPS	Mpn	+	+	+

21	F	13	NPS	Mpn	+	+	+

22	M	47	Sputum	Mpn	+	+	+

23	F	14	NPS	Mpn	+	+	+

24	F	5	NPS	Mpn	+	+	+

25	F	70	Sputum	Mpn	+	+	+

26	F	5	NPS	Mpn	+	+	+

27	M	44	Sputum	Mpn	+	+	+

28	F	43	TS	Mpn	+	+	+

29	M	4	Sputum	Mpn	+	+	+

30	F	7	TS	Mpn	+	+	+

31	M	5	TS	Mpn	+	+	+

32	M	15	ETA	Mpn	+	+	+

33	M	10	TS	Mpn	+	+	+

34	M	8	TS	Mpn	+	+	+

35	F	23	Pharyngeal	Mpn	+	+	+

36	M	50	TS	Mpn	+	+	+

37	F	7	BAL	Mpn	+	+	+

38	NA	NA	NA	Mpn	+	+	+

39	F	61	Sputum	Mpn	+	-	-

40	F	7	NPS	Mpn	+	-	-

41	M	6	NPS	Mpn	+	-	-

42	M	18	TS	Mpn	+	-	-

43	M	9	NPS	Mpn	-	+	-

*44	NA	NA	NPS			+	+

*45	NA	NA	NPS			+	+

*46	NA	NA	NPS			+	+

DNA for "home-brew"monoplex real-time PCR was extracted from specimens using the MagNA Pure LC (Roche, Mannheim, Germany) as per manufacturer's instructions. Specimens were tested using a "home-brew" single target monoplex real-time PCR assay [12]. Real-time amplification was carried out using the Roche Light Cycler (Roche, Mannheim, Germany), as previously published [12,13].

### Mycoplsma pneumoniae

Clinical specimens from patients with suspected *M. pneumoniae *infection were cultured in PPLO medium containing thallium acetate, horse serum, yeast dialysate and supplemented with amphotericin B (0.5 mg/ml), penicillin G (100,000 u/ml) and nystatin (50,000 u/ml). Biphasic culture flasks were inoculated with specimens, incubated at 37°C and inspected daily for 4 weeks. The organism was identified based on typical colonial morphology on the agar medium and the change in the broth color from red to orange then to yellow in the absence of turbidity of the broth.

### ProPneumo-1 and PneumoBacter-1 assays

Nucleic acid, for analysis by the ProPneumo-1 (Prodesse, Waukesha, WI) or the PneumoBacter-1 (Seegene, Korea) assay, was extracted using the easyMag NucliSense magnetic extraction (bioMerieux, Netherlands) [14]. Each nucleic acid extract was amplified by both, the PromPneumo-1 and PneumoBacter-1 multiplex PCR assays. The ProPneumo-1 assay was performed as per manufacturer's instructions. Amplification and detection of the ProPneumo-1 test was performed on the ABI 7500 (Applied Biosystems, Foster City, CA). For discordant analysis all specimens, including concordant specimens, were re-tested using the PneumoBacter-1 assay (Seegene, Korea). PneumoBacter-1 is an agarose gel-based assay, amplification was performed on the iCycler (Bio-Rad, Milpitas, CA) and amplicons were imaged, following gel electrophoresis, as per manufacturer's protocols.

### Experimental controls: external, internal and extraction controls

*M. pneumoniae *and *C. pneumoniae *quantified DNA controls (Advanced Biotechnologies Inc., Columbia, MD) were included in each assay as external positive controls. For internal DNA control and to monitor for potential PCR inhibitors, exogenous DNA was spiked into each specimen. To monitor for the integrity of extracted nucleic acid, all specimens were also tested for the presence of the housekeeping gene, human glyceraldehyde phosphate dehydrogenase (GAPDH) (Applied Biosystems. Foster City, CA). Real-time amplification and detection of the GAPDH gene sequence was performed on the ABI 7900HT (Applied Biosystems, Foster City, CA), as previously described [15,16]. This test was required to ascertain the presence of nucleic acid in specimens, which tested negative for *C. pneumoniae *or *M. pneumoniae*. For negative controls, target nucleic acid and specimens were replaced by PCR grade water in each PCR assay or nucleic acid extraction run, respectively.

### Limits of detection

The limits of detection for the assays described in this study, ProPneumo-1 and PneumoBacter-1, were determined using 10 fold serial dilutions of quantified *M. pneumoniae *and *C. pneumoniae *DNA (Advanced Biotechnologies Inc., Columbia, MD) in PCR-grade water. Data analysis was carried out using SPSS 15.0 (SSPS Inc., Chicago, IL). Turn-around-times per assay were estimated from a review of laboratory procedures and include time from specimen reception to results reporting.

### Definitions

The following definitions were used for analysis. **Concordant specimen**: agreement between "home-brew" assay result and Prodesse ProPneumo-1 assay result. **Discordant specimen**: disagreement between "home-brew" assay result and Prodesse ProPneumo-1 assay result. **Resolved specimen**: agreement in result, either positive or negative between any two of the following assays; 1) "home-brew" or culture, 2) ProPneumo-1, and 3) PneumoBacter-1.

## Results

Table 1 summarizes the results obtained for the evaluation of the ProPneumo-1 real time multiplex PCR assay. Overviews of the results obtained for subsets of *M. pneu*moniae and *C. pneumoniae *patients are presented in Table 2 and Table 3, respectively. Among patients who tested positive for either pathogen, the ratio of male to female is approximately the same and, with the majority of positive patients being under the age of 20 years. Table 2 also displays the results obtained for each of the three tests used to identify positive *M. pneumoniae *(A) or *C. pneumoniae *(B) patients. Using a monoplex "home-brew" real-time PCR as a reference in the analysis of *C. pneumoniae *and culture as a reference in the analysis of *M. pneum*oniae, 14 and 41 specimens tested positive for *C. pneumoniae *and *M. pneumoniae*, respectively by the ProPneumo-1 assay. As shown in Table 1, 11 patients tested positive for *C. pneumoniae *by the "home-brew" monoplex assay and 42 patient*s *tested positive for *M. pneumoniae *by culture. Similarly, 48 and 43 specimens were defined as negative in the ProPneumo-1 assay, for *M. pneumoniae *and *C. pneumoniae*, respectively. Although, 42 positive *M. pneumoniae *specimens were identified by culture and 41 by the ProPneumo-1assay, the identity of 8 positive specimens however, varies between the two methods (see Table 2). Curiously, both assays the ProPneumo-1 and PneumoBacter-1 detected 5 additional positive specimens, 3 for *M. pneum*oniae and 2 for *C. pneumoniae *in the *C. pneumoniae*-negative and *M. pneumoniae*-negative pool, respectively.

**Table 3:** Overview of test results for a subset of *C. pneumoniae *patients

**Patient No**	**Sex**	**Age**	**Specimen**	**Pathogen**	**Monoplex** **PCR**	**Prodesse** **PP-1**	**Seegene** **PB-1**
01	M	13	BAL	Cpn	+	+	+

02	M	0.2	NPS	Cpn	+	+	+

03	F	10	BAL	Cpn	+	+	+

04	F	10	ETA	Cpn	+	+	+

05	F	2	BAL	Cpn	+	+	+

06	F	10	BAL	Cpn	+	+	+

07	F	63	NPS	Cpn	+	+	+

08	M	32	NPS	Cpn	+	+	+

09	F	63	TS	Cpn	+	+	+

10	F	19	NA	Cpn	+	+	+

11	M	48	BAL	Cpn	+	-	-

12	NA	NA	Autopsy	Cpn	-	+	+

13	NA	NA	Sputum	Cpn	-	+	+

**14	F	25	NPS			+	+

**15	F	44	NPS			+	+

Abbreviations: NA, not available; M, male; F, Female; NPS, nasopharyngeal swab; NPA, nasopharyngeal aspirate; BAL, bronchoalveolar lavage; ETA, endotracheal aspirate; TS, throat swab; BR, bronchial; Cpn, *C. pneumoniae*; Mpn, *M. pneumoniae*; PP-1, ProPneumo-1; PB-1, PneumoBacter-1. *, These specimens tested negative for *C. pneumoniae *in the monoplex assay but were defined positive for *M. pneumoniae *by the ProPneumo-1 and PneumoBacter-1 assays. **, These specimens tested negative for *M. pneumoniae *in culture but were defined positive for *C. pneumoniae *by the ProPneumo-1 and PneumoBacter-1 assays.

The limit of detection of the ProPneumo-1 assay as determined by Probit regression (95% probability) was estimated as follows: 1 genome equivalent (Geq)/reaction for *M. pneumoniae *and 1 Geq/reaction for *C. pneumoniae*. The limit of detection for PneumoBacter-1 was 1 Geq/reaction for *M. pneumoniae *and 1 Geq/reaction for *C. pneumoniae*. The turn-around-times, from specimen reception to reporting, are as follows: ProPneumo-1, 6 hours; "home-brew" monoplex PCR, 10 hours; PneumoBacter-1, 12 hours, and Mycoplasma culture, 4 weeks.

### Diagnostic specificity and sensitivity

To determine the diagnostic specificity and sensitivity of the ProPneumo-1 assay, results were scored against the reference method ["home-brew" monoplex real-time PCR for *C. pneumoniae *(see Table 4) and culture for *M. pneumoniae *(Table 5)]. To resolve discordant results, specimens were re-tested using the multiplex PneumoBacter-1 method (Tables 4 and Table 5).

**Table 4:** Specificity and sensitivity of the ProPneumo-1 assay for *C. pneumoniae*.

Before discordant analysis			
***C. pneumoniae***	**Monoplex "Home-Brew" Results**		

**ProPneumo-1 Results**		Positive	Negative
	
	Positive	10	2
	
	Negative	1	45
	
	Total	10/11	45/47
	
		Sensitivity = 91%	Specificity = 96%

Following discordant analysis with PneumoBacter-1			

***C. pneumoniae***	**Reference Method Results**		

**ProPneumo-1 Results**		Positive	Negative
	
	Positive	14	0
	
	Negative	0	43
	
	Total	14/14	43/43
	
		Sensitivity = 100%	Specificity = 100%

**Table 5:** Specificity and sensitivity of the ProPneumo-1 assay for *M. pneumoniae*,

Before discordant analysis			
***M. pneumoniae***	**Culture Results**		

**ProPneumo-1 Results**		Positive	Negative
	
	Positive	38	1
	
	Negative	2	47
	
	Total	38/40	47/48
	
		Sensitivity = 95%	Specificity = 98%

Following discordant analysis with PneumoBacter-1			

***M. pneumoniae***	**Reference Method Results**		

**ProPneumo-1 Results**		Positive	Negative
	
	Positive	41	1
	
	Negative	0	47
	
	Total	41/41	47/48
	
		Sensitivity = 100%	Specificity = 98%

The ProPneumo-1 assay detected 14 positive *C. pneumoniae *specimens compared to 11 in the monoplex "home-brew" assay, of which 1 specimen was false positive. Similarly the ProPneumo-1 detected 43 negative *C. pneumoniae *specimens compared to 47 in the monoplex "home-brew" assay, of which 2 specimens were false negative (Table 1 and Table 3). Prior to discordant analysis, the sensitivity and specificity of the ProPneumo-1 assay for *C. pneumoniae *was 91% and 96%, respectively (Table 4).

In the case of *M. pneumoniae*, ProPneumo-1 and culture detected 42 and 41 positive specimens, respectively. However, 4 specimens scored positive by the culture method (Table 1 and Table 2) and negative by the PCR method. Likely, due to repeated freeze-thaw cycles and the long storage period at -80°C (up to 10 years), these 4 specimens are devoid of intact bacterial DNA and yielded negative results in both PCR tests. Similarly, one specimen appeared false positive by the ProPneumo-1 assay. We suggest that this specimen is likely a true positive which was not detected by the PneumoBacter-1 assay, likely due to its gel-based format.

Prior to discordant analysis the sensitivity and specificity of the ProPneumo-1 assay for *M. pneumoniae *was 95% and 98%, respectively (Table 5). Following discordant analysis by the PneumoBacter-1 multiplex assay and classification of specimens as per the definitions described above, the sensitivity and specificity of the ProPneumo-1 assay for *C. pneumoniae *was 100% and 100%, respectively (Table 4). The sensitivity and specificity of the ProPneumo-1 assay for *M. pneumoniae*, however, was 100% and 98%, respectively (Table 5). Consistent with its multiplex format, the ProPneumo-1 assay detected 5 additional positive specimens, 2 *C. pneumoniae *in the *M. pneumoniae*-negative pool and 3 *M. pneumoniae *in the *C. pneumoniae*-negative pool.

## Discussion

Early work in molecular diagnostics utilized technologies that were considered to be site-developed or "home-brew" in nature. Although these assays were considerable advances at their time, they were often plagued by requirements for up-front research and development, quality assurance, and laboratory accreditation issues [10,11]. As a result, past molecular diagnostics for the clinical microbiology laboratory were often restricted to laboratories that had the monetary and human resources to meet these demands. In the last five years however, commercial products such as those identified in this manuscript have allowed for the implementation of molecular diagnostics without the up-front requirement for research and development as well as simplified quality assurance steps.

The ProPneumo-1 assay demonstrated several strengths as a commercial product for use in a wider range of clinical microbiology laboratory settings outside of the reference or highly resourced setting. First, this assay is a rapid, sensitive and specific method that allows for the diagnosis of *C. pneumoniae *and *M. pneumoniae *in a variety of clinical specimens. Second, the fact that the Pro-Pneumo-1 assay is a commercial product allows for simpler implementation in the diagnostic laboratory, and continued validation (e.g. quality assurance) when compared to "home-brew" assays [10,11]. Third, the ProPneumo-1 assay is a multiplex assay, which allows for multiple primer combinations in one reaction, instead of multiple monoplex reactions. This not only makes the assay easier to perform in a laboratory but also has the potential of utilizing less patient specimen [17]. Indeed, using the ProPneumo-1 multiplex assay we detected 5 additional positive specimens, 2 *C. pneumoniae *in the *M. pneumoniae*-negative pool and 3 *M. pneumoniae *in the *C. pneumoniae*-negative pool.

There are several limitations to this study including its retrospective nature. A further limitation of this study is the relatively low number of *C. pneumonia*-positive specimens analyzed. However, the number of specimens analyzed represents the total complement of specimens available from this region over a period of ten years. The authors hope that further prospective analysis will allow for the further characterization of this commercial assay.

Several studies have evaluated the use of multiplex PCR for the detection of *M. pneumoniae *and *C. pneumoniae *in clinical specimens [18-20]. These studies have used a variety of assays, patient populations and sample types, thus proper validation and standardization are often lacking. These factors make it difficult to compare different studies although the finding has been generally consistent. A study by Khanna and colleagues [19] described the Pneumoplex assays (standard and real-time PCR) that can detect 5 pathogens, including *M. pneumoniae *and *C. pneumoniae*, in a single reaction. The diagnostic sensitivity of the Pneumoplex assay was 100% and was defined by spiking negative BAL specimens with bacterial DNA. The diagnostic specificity and sensitivity of the ProPneumo-1 assay, on the other hand, were determined in this study directly from patient's specimens. Another study by Ginevra and colleagues [20] described the triplex Chlamylege assay, for the detection of *Legionella*, *M. pneumoniae *and *C. pneumoniae*. The diagnostic specificity of the Chlamylege assay was also 100% and it was defined by sequencing the DNA of discordant specimens. Compared to our ProPneumo-1 study, fewer positive specimens were used for the evaluation of the Chlamylege assay (2 *C. pneumoniae *and 9 *M. pneumoniae*). However, the lack of an of appropriate "gold standard" for the quantitative analysis of intracellular pathogens makes comparisons of the sensitivities of these different assays difficult.

In conclusion, the ProPneumo-1 real-time multiplex PCR assay is a sensitive, specific, convenient and reliable tool for the detection of atypical respiratory infection pathogens directly from respiratory tract specimens. The authors believe that this product can be effectively implemented in a wide variety of clinical microbiology settings outside of reference and highly resourced laboratory settings.

## Competing interests

The authors declare that they have no competing interests.

## Authors' contributions

RRH undertook data analysis, assisted in validation study planning and drafted a manuscript. EL and PT participated in validation study planning and work. KR, AM, and SB undertook specimen collection and reference method analysis. FJ assisted in planning of experiments. SJD planned and coordinated study and drafted manuscript.
